# Enhancing the Internet of Things with Knowledge-Driven Software-Defined Networking Technology: Future Perspectives

**DOI:** 10.3390/s20123459

**Published:** 2020-06-19

**Authors:** Yuhong Li, Xiang Su, Aaron Yi Ding, Anders Lindgren, Xiaoli Liu, Christian Prehofer, Jukka Riekki, Rahim Rahmani, Sasu Tarkoma, Pan Hui

**Affiliations:** 1 State Key Laboratory of Networking and Switching Technology, Beijing University of Posts and Telecommunications, Beijing 100876, China; 2 Department of Computer and Systems Sciences, Stockholm University, 16407 Stockholm, Sweden; rahim@dsv.su.se; 3 Department of Computer Science, University of Helsinki, FI-00014 Helsinki, Finland; xiang.su@helsinki.fi (X.S.); xiaoli.liu@helsinki.fi (X.L.); sasu.tarkoma@helsinki.fi (S.T.); pan.hui@helsinki.fi (P.H.); 4 Center for Ubiquitous Computing, University of Oulu, FI-90014 Oulu, Finland; jukka.riekki@oulu.fi; 5 Department Engineering Systems and Services, Delft University of Technology, 2628BX Delft, The Netherlands; Aaron.Ding@tudelft.nl; 6 RISE Research Institutes of Sweden, 16440 Kista, Sweden; anders.lindgren@ri.se; 7 Luleå University of Technology, 97187 Luleå, Sweden; 8 DENSO Automotive Germany GmbH, 85386 Eching, Germany; christian.prehofer@tum.de; 9 Department of Informatics, Technical University of Munich, 80333 München, Germany; 10 Department of Computer Science and Engineering, The Hong Kong University of Science and Technology, Clear Water Bay, Hong Kong

**Keywords:** Internet of Things (IoT), Software-defined Networking (SDN), knowledge-driving networking, IoT-proxy

## Abstract

The Internet of Things (IoT) connects smart devices to enable various intelligent services. The deployment of IoT encounters several challenges, such as difficulties in controlling and managing IoT applications and networks, problems in programming existing IoT devices, long service provisioning time, underused resources, as well as complexity, isolation and scalability, among others. One fundamental concern is that current IoT networks lack flexibility and intelligence. A network-wide flexible control and management are missing in IoT networks. In addition, huge numbers of devices and large amounts of data are involved in IoT, but none of them have been tuned for supporting network management and control. In this paper, we argue that Software-defined Networking (SDN) together with the data generated by IoT applications can enhance the control and management of IoT in terms of flexibility and intelligence. We present a review for the evolution of SDN and IoT and analyze the benefits and challenges brought by the integration of SDN and IoT with the help of IoT data. We discuss the perspectives of knowledge-driven SDN for IoT through a new IoT architecture and illustrate how to realize Industry IoT by using the architecture. We also highlight the challenges and future research works toward realizing IoT with the knowledge-driven SDN.

## 1. Introduction

Internet of Things (IoT) ubiquitously connects identifiable and addressable devices with limited storage, processing, and networking capacities. The development of IoT is accelerated by advances in electronics, sensing, communications, networking, and big data technologies. Various IoT devices and applications are developed for collecting data and performing tasks for different domains, ranging from environmental monitoring, industrial process systems, surveillance, traffic, and disaster monitoring, to a large variety of end user applications.

The development of IoT has resulted in large-scale IoT networks with vast numbers of heterogeneous devices, which are facing the following problems. (i) Difficulties in control and management. IoT applications serve different purposes and are deployed in isolated ways. Heterogeneous devices are geographically distributed and used in various application domains. (ii) Difficult to program and configure the devices. On account of the huge difference of devices’ capabilities, especially the constrains in memory, bandwidth and energy, it is difficult to program or configure the devices with new functions in a unified and efficient way. (iii) Long service provisioning time. The deployment of a new IoT service requires the whole cycle of developing the new service, including installing new sensors, setting up connections to the network infrastructure, and testing the functions. (iv) Resources have not been fully used. Data and devices have not yet been considered to be network resources. Moreover, scalability, flexibility, complexity, security as well as efficient data, traffic and device management are also challenges for the IoT networks. An essential reason behind these problems is that the IoT networks lack flexibility, intelligence, and application-specific controls.

Software-defined networking (SDN) technology is characterized by separating the control and data plane, providing programmability and standardized APIs. SDN enables a global view of the network and provides capabilities to use network resources efficiently. Therefore, SDN reduces the overhead of network management and improves the flexibility of networks, which presents a great potential to solve or mitigate the emerging problems of IoT. Some work has been done to introduce SDN into IoT, as listed in Section 3 and surveyed in [1,2,3]. However, most research focuses on using SDN to achieve only specific improvements to IoT. Few researchers have discussed the requirements on SDN caused by IoT systems or new issues raised by the SDN-based IoT environments. Although SDN simplifies the control and management of both IoT networks and applications, SDN is not specifically designed for IoT. Hence, critical challenges have to be addressed before SDN can be used in IoT environments.

IoT collects data from connected devices, which will be analyzed and used in time by various applications. However, IoT data has not been typically used for improving the intelligence and flexibility of the IoT network itself. In this paper, we argue of the enhancement of IoT through SDN, with a focus on using data from IoT itself. We survey the scattered work related to data for SDN and IoT. To the best of our knowledge, although some work has been done to introduce data, knowledge and SDN to IoT, as reviewed in Section 4.2, there is no work discussing knowledge-driven SDN for IoT, considering the features and development of IoT. To well explain future perspectives of IoT, we introduce a knowledge-driven SDN-based IoT architecture to illustrate how the flexibility and intelligence of the IoT networks can be enhanced, how the application-specific control can be realized, and the crucial challenges of current IoT networks can be addressed. We highlight also the challenges toward enhancing IoT with knowledge-driven SDN.

The remainder of the paper is organized as follows. In Section 2, we review the evolution of SDN and characteristics of IoT. Then, we summarize the benefits brought by SDN to IoT, identity the challenges when integrating SDN into IoT, and analyze the problems needed to be solved with the help of IoT data in Section 3. Based on this, in Section 4 we review the current research related to data-driven SDN for IoT, illustrate the perspectives of IoT enhanced by knowledge-driven SDN-based IoT architecture, elaborate how the problems faced by IoT can be addressed, and present an example application of Industry IoT. Finally, we highlight the research work toward enhancing IoT with knowledge-driven SDN in Section 5 and conclude the paper in Section 6.

## 2. SDN and IoT Reviews

### 2.1. SDN and Its Evolution in Wireless and IoT Networks

**Figure 1:**
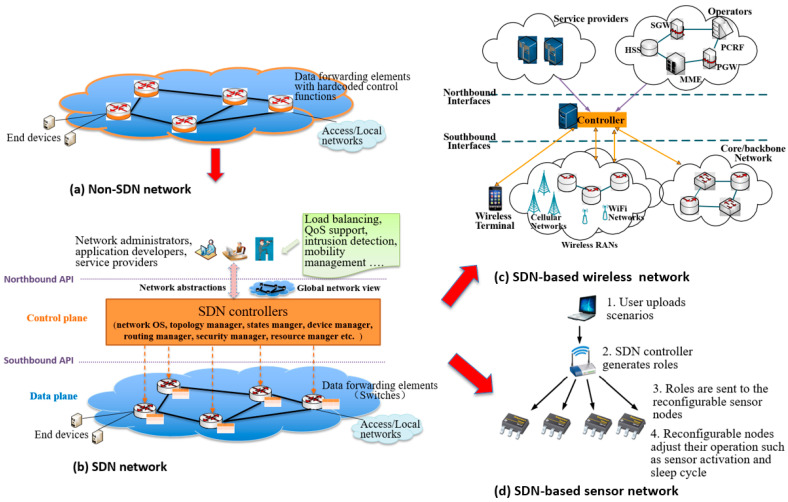
From non-SDN to SDN, then to Software-defined Wireless Network and Software-defined Sensor Networks. (**a**) a non-SDN network, where data forwarding elements (i.e., routers) have hard-coded control functions (e.g., forwarding policies). (**b**) an SDN network, where forwarding policies of the data forwarding elements (i.e., switches) can be controlled (configured) by logically centralized SDN controllers through standard APIs. (**c**) SDN is used in a wireless network where a centralized SDN controller owns all the network information to optimize resource usage and network load among heterogeneous RANs. (**d**) SDN is used in a sensor network with an aggregator (sink) node as a controller that implements device-based control and optimization, such as duty cycles.

The basic idea of SDN is to decouple network control and data forwarding functions (Figure 1a) into control and data planes and provide standard APIs in order to improve the programmability of networks [4,5,6]. SDN enables networking elements (i.e., switches in Figure 1b) on the data plane to be dynamically configured by the controllers on the control plane. New services can be programmed and injected into the SDN controllers through a standard northbound API, which correspondingly configures the routing tables of the switches (i.e., flow tables), guiding the switches to forward the packets or flows of the user applications through a standard southbound API. SDN benefits networks with:

- A global view of the whole network, including its resources. Hence, network resources can be used more efficiently.
- Reduced overhead of network management, due to software-configured devices and network resources that expose uniform interfaces through standard abstractions.
- Improved network flexibility through programmability, i.e., new network services can be provided on the fly through standard APIs and network function abstractions.

SDN was originally designed for the core network and data centers, where highly capable switches/routers are used which the main targets are to optimize the usage of bandwidth and other resources. Recently, SDN has been applied to wireless cellular networks, where several heterogeneous Radio Access Networks (RANs) coexist [7], as shown in Figure 1c. This architecture enables the joint optimization of RAN and core backbone resources to improve quality of service (QoS) and extends programmability from service providers to users, allowing quick and simple service deployment and adaptation and fine-grained mobility management.

Compared with cellular networks, IoT networks are heterogeneous and decentralized. With the increased number of IoT applications and devices, controlling the network and devices becomes more complex. This has provided a new domain where SDN can play a role. Figure 1d presents an architecture for SDN-assisted sensor network [8]. The SDN controller is implemented at a central sink or aggregator node, making it suitable for topology and device-based control. Furthermore, the controller optimizes the routing according to application requirements based on its holistic network view. The SDN controller can also choose which sensor to activate if a node has multiple sensors satisfying the requirements of applications. This allows sensor nodes to be used by multiple applications without redesign, leading to a more sustainable and incrementally growing sensor and service ecosystem.

Nevertheless, the integration of SDN and wireless technologies is not straightforward. Typical networking technologies are not compatible with SDN standards such as OpenFlow. In addition, SDN mainly focuses on controlling traffic flows and lacks the capability of controlling sensor hardware and IoT applications.

### 2.2. IoT and Its Characteristics

IoT systems have the major task of collecting data from environments and making the data accessible and usable when needed. Compared with traditional Internet services, IoT differentiates in the following aspects [9,10,11]:

**Large amounts of devices and data.** The large amount of connected IoT devices generate huge amounts of data which the network must handle in a scalable manner. Data needs to be either continuously uploaded to a central cloud, which may cause heavy upload traffic; or processed locally, which may require certain local computational resources and distributed algorithms.

**Heterogeneity of devices, data, and services.** Networks and connected devices are heterogeneous in terms of capabilities, capacity, and the amount and type of data generated. Individual nodes can generate large amounts of data while not possessing large computational capability. Other nodes only produce small triggered updates. Similarly, application requirements often state that services must have low computational complexity, high energy efficiency, low latency or high bandwidth to the cloud.

**Application and QoS requirements.** Internet applications have a limited set of requirements, such as high-quality video, audio, and variable data rates. IoT presents an application dependent set of requirements. For example, remote operation for e-health requires large bandwidth and low latency, but remote metering of water requires low bandwidth and can endure high latency.

**Resource constraints and network connectivity.** Most IoT devices and networks are more resource constrained than those for traditional Internet services. Cheap and energy efficient devices as well as low-power and low bandwidth communication channels enable large and long-term deployments operating independently without a continuous power supply. IoT devices may also be intermittently disconnected from the rest of the network, because of varying network conditions or duty-cycling of devices for power saving.

**Isolated islands of deployment.** Many IoT deployments are closed local systems where most communication happens within an isolated domain. The reason can be a lack of open IP-based connectivity, reliance on proprietary communication solutions between devices, or a desire to keep the system protected from outside access. At the same time, some stakeholders can request communication with other parts of the network to access more data, for remote control, or to provide selected data for an open ecosystem.

**Security and privacy [12,13].** Data collected by IoT devices is often sensitive as it can expose personal information about the user or people nearby. IoT services may even allow certain physical devices to be controlled. Therefore, it is important to protect access to IoT data and services. However, constrained devices may not have the necessary computational power to carry out the required cryptographic operations. Moreover, uncoordinated deployments and network disconnections make it difficult to maintain contact with security infrastructure, which prevents IoT devices from making initial key setup and the establishment of identities.

The above characteristics of IoT put forward challenges to create a fast, secure, and efficient network infrastructure. Addressing these challenges requires new ways of applying SDN technology as compared with its current use in datacenters and core networks.

## 3. SDN for IoT

### 3.1. Benefits of SDN to IoT

Introducing SDN to IoT involves architecture, protocols, interfaces, management, etc., which has been surveyed in some publications. Table 1 lists a comparison of the surveys, most of which concentrate on various solutions of how to introduce SDN to IoT. In the following discussion, we concentrate on reviewing how IoT and which metrics of IoT can be improved by using SDN.

Due to the characteristics of IoT, it is difficult to manage and control the IoT applications, devices, and data in a general and scalable way. In particular, deploying new IoT applications will take a long time and the whole network resources cannot be used efficiently.

SDN empowers easy management and network programmability. The underlying network infrastructure and resources are abstracted for applications and network services, which brings significant benefits to IoT. For instance, SDN may reduce the complexity of IoT network management and control. IoT devices are normally deployed at the edge of networks. Network equipment, such as sinks, gateways, and servers are needed to collect and process data, which increases the complexity of the network considerably. SDN makes it possible to overcome the complexity by rapidly configuring and directly programming the network equipment and devices. In addition, SDN can guarantee and improve the performance of IoT services through dynamic resource management, such as load balancing and bandwidth scheduling, etc. SDN can also provide prompt network services for IoT. For example, SDN can create a virtual chain of services for IoT, which handles specific traffic for specific IoT devices or applications. SDN may increase the security of IoT environments and networks by, e.g., segmenting the network flexibly and limiting the impact caused by any breaches from any IoT device [23,24]. Furthermore, SDN may also reduce the management cost of IoT network and optimize networks specifically for IoT. Table 2 illustrates the benefits of SDN to IoT with the examples of practices.

### 3.2. Challenges When Integrating SDN and IoT

Some previous research has been done to improve IoT by using SDN. However, these efforts have only solved specific problems encountered during the development of IoT and SDN, case by case. The integration of SDN and IoT has not been considered to be a whole to solve general problems. In particular, the following issues should be considered before introducing SDN into IoT.

First, SDN has its own weaknesses stemming from the central control and limited routing table sizes of switches. To match the demands from IoT, the weakness of SDN itself should be overcome, such as scalability and single point of failure.

Second, IoT provides a new application environment for SDN. However, SDN needs to be enhanced in order to perform the management and configuration tasks efficiently that IoT requires. In Table 3, we highlight the techniques used by SDN and identify the corresponding problems that may occur when SDN is used in the IoT environment and sketch the requirements set to SDN. Here it is worth noting that IoT puts a special requirement on the placement of SDN controllers due to the requirement of large amount data processing and large numbers of heterogeneous IoT devices.

Third, efficient and reliable identification schemes for IoT devices and flexible definition of their networking interfaces are the basis for integration of SDN and IoT. For instance, SDN needs to cope with special hardware, possibly through an abstract interface.

Moreover, efficient methods for calculating and evaluating the influence of IoT traffic on the network infrastructure are also needed. Also, when SDN is used to support IoT related networking elements, additional security concerns should be addressed.

Hence, in order to enhance IoT by making use of the benefits brought by SDN, SDN should be adapted to satisfy the requirements from IoT. Moreover, due to the unique characteristics of IoT, the applications and data of IoT should also be considered in the SDN-based environment.

## 4. Perspectives of Knowledge-Driven SDN for IoT

### 4.1. Knowledge for SDN and IoT

SDN has the potential to optimize network usage, improve the performance of applications, and introduce new services and protocols in the network, rapidly and flexibly. Nevertheless, all such potentials are based on the intelligence (i.e., decision-making) of the SDN controller, which are eventually decided by the network-wide information available to the controller. However, the SDN controller typically only uses the information captured by the network through traffic measuring and analysis in a small scale in the current SDN-based network solutions. The current SDN network architecture is not designed to use data monitored from the global or large-scale network infrastructure, or related with the results of various applications running in the network to enhance the controlling functions. Undoubtedly, this limits the intelligence and flexibility of the SDN network.

In the context of IoT, the following aspects need to be improved with the intelligence in the network.

- **Application-specific control.** Both IoT applications and the corresponding IoT standards are domain-dependent. Currently, there are neither cross-industrial standards nor reference designs. Besides, the interoperability at different levels of software and hardware is difficult to realize. These may cause the resources in the network to be underused and increase the networking cost.
- **Device and data management.** Management of hardware, software and services needs to be kept in pace with the increasing number of devices and data. In particular, real-time events should be handled effectively.
- **Collective intelligence.** Individual nodes and sensors may not be smart enough to provide smart services at low prices. Thus, there is a strong demand to use artificial intelligence, semantics, and device clusters to bring in collective intelligence.
- **Software, services, and algorithms.** Although many IoT applications and infrastructures have been developed for different industrial domains, the basic problems of lacking open IP-based connectivity and the slow IPv6 deployment make it difficult to realize low-power software, services and algorithms that support heterogeneous nodes in order to build a self-managing and self-healing IoT network.
- **Security and privacy.** Security and privacy of IoT are important topics, which need further investigation. Authorized access, privacy of user data, models for decentralized authentication, and integrity on consumer devices are still missing.

Centralized management and control with sufficient network-wide information and knowledge can help to solve these problems. The shared data and knowledge obtained from individual vertical application domains can greatly promote the realization of the horizontal deployment of IoT, and make full use of various kinds of resources. The efficient, scalable, and high level of data abstraction and analysis can help to manage the heterogeneous device and data. Efficient coordination and control of data across devices can help to realize collective intelligence and flexible and scalable deployment of various services, algorithms, efficient protocols and security and privacy related mechanisms.

### 4.2. State of the Art of Knowledge for SDN and IoT

Due to the importance of knowledge to SDN and IoT, some work has already been done in dealing with data or knowledge for SDN and IoT.

Work [54] suggests an SDN-based IoT architecture solving the problem of large amounts of data generated in IoT. Instead of evaluating the data from sensors in the application layer, Ref. [54] analyzes them in lower layers, especially in the gateway layer, before being sent to the Internet. The proposed architecture concentrates on solving the big data problems of IoT with the help of SDN. However, the data has not been used to assist the decision-making for SDN in order to efficiently serve IoT functions. Issues such as application-specific control, collective intelligence, security, privacy, etc., that need to be improved in IoT were not discussed. [55] proposes a collaborative edge-cloud computing platform for IoT data analytics. The global view of SDN is applied to control the states of the virtual computing, storage, and communications resources of the overall system, and allocate resources in edge or cloud nodes to run the machine learning algorithms to analyze the heterogeneous IoT data. Similar to [55,56] also deals with the efficient distribution of IoT analytics among the core and edge network with the help of SDN. It uses SDN to deploy the IoT traffic control and congestion avoidance mechanisms to perform dynamic distribution of IoT processing among the edge and cloud nodes based on the network resource states. Though the above two works involves the application-control of IoT, other issues have not been discussed.

Using data in the network has the potential to increase the intelligence and flexibility of networks. Work [57] suggests a data-driven information plane to SDN networks which emphasizes the role of a variety of network data originating from the infrastructure to promote network intelligence and maximize its inherent value in network design, configuration, management, and programmability. Although [57] suggests a general data-driven SDN architecture without mentioning IoT, it provides an idea of the future development of SDN. This will be helpful for designing the future IoT network. Similarly, work [58] suggests a data-driven intelligent future network architecture, where a big data engine is integrated in the control plane of SDN. Data processing, data analysis and decision support are involved in the big data engine. Although the network architecture in [58] is designed for dealing with the content delivery in the future Internet, the data in the big data engine can be obtained from both network data and application data. This can benefit also the future IoT networks.

Table 4 summarizes the above work.

Currently, machine learning (Artificial Intelligence) has been used in cloud and edge/fog computing in the context of IoT for analysis of data and to implement certain control functions [59,60]. For example, data forwarding and resource allocation functions at edge nodes can be adapted according to the results of data analysis at the edge nodes. However, the intelligence and control in these systems are different from the knowledge-driven SDN for IoT, see Table 5. One of the major differences is that the intelligence of knowledge-driven SDN for IoT is distributed in different network entities—the SDN controllers, IoT controllers, IoT proxies etc. Even the data forwarding devices such as gateways can have certain level of intelligence. In this way, the intelligence of knowledge-driven SDN can be scalable to IoT devices compared with that of cloud and edge computing with AI, where the intelligence is located only in a cloud server or edge nodes. In addition, the intelligence of current cloud or edge computing with AI is generally used for supporting the decision-making for various applications and devices. However, the intelligence of knowledge-driven SDN for IoT is generally used for control and management of different levels of IoT networks, applications, and devices.

Data fusion is an example that the knowledge-driven SDN technique can benefit the IoT systems. In the field of IoT, data fusion is a common technique for handling multiple data sources [61], which can improve the quality of data output or extract knowledge from the raw data. Currently, research is focused on using machine learning methods, including the deep neural network, unsupervised data fusion and hybrid models to fuse wide variety of data sources. Typically, data fusion is implemented on the edge computation platform [62], fog computation platform [63], cloud computation platform [64,65] or a hybrid computation platform where the processing is realized at both edge or cloud [66]. However, by using the method described in Section 4.3, various data fusion algorithms can be distributed at sinks or gateways, and the results can be sent to the knowledge plane, where new knowledge can be further extracted by using the knowledge in the knowledge plane. Also, the extracted knowledge cannot only be sent back to the applications, but also be used to guide more gateways or sinks to collect different raw data or in a different way through different levels of controllers.

### 4.3. A Knowledge-Driven SDN-Based Architecture for IoT: Future Perspective

Although some work has been done to introduce knowledge and SDN to IoT, as reviewed in Section 4.2, there is no work discussing knowledge-driven SDN for IoT, considering the features and development of IoT. To show the importance and feasibility of knowledge and SDN for IoT and to analyze the perspectives of knowledge-driven SDN-based IoT, we suggest a knowledge-driven SDN-based IoT (KN-SDN-IoT) architecture, which considers the unique characteristics of IoT discussed in Section 2.2. KN-SDN-IoT introduces a knowledge plane, which makes full use of IoT application and network operation data compared with the traditional SDN-based networks. It also introduces IoT specific functionalities to the control plane. The control logic can be IoT application specific, supporting heterogeneous IoT systems. The programmability brought by SDN and the knowledge brought by IoT data enable both flexibility and intelligence to the IoT system, making it an autonomous system.

**Figure 2:**
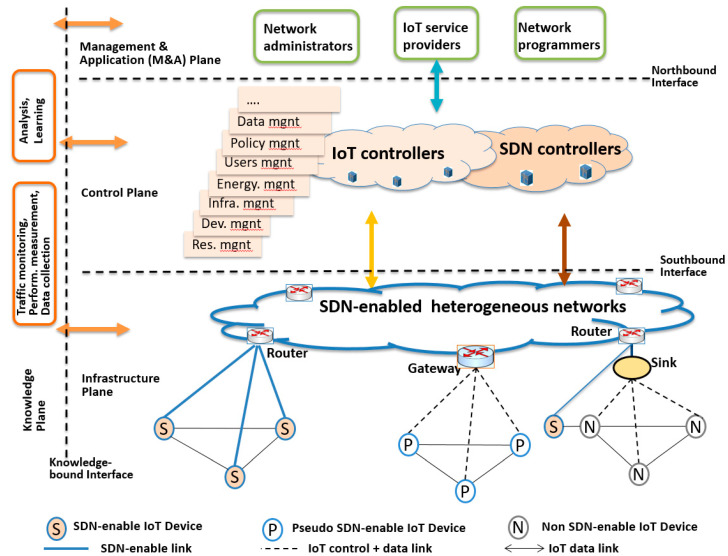
A knowledge-driven SDN-based IoT architecture.

Figure 2 shows the architecture that consists of four planes: the management and application-specific service (M&A) plane, control plane, infrastructure plane, and knowledge plane. IoT service providers and network administrators program various network services through the M&A plane. Policies, strategies, algorithms, and service logics can be programmed to IoT controllers in the control plane through the standardized northbound interface.

An SDN controller and multiple IoT controllers construct the control plane. The SDN controller is responsible for running the network in an optimized manner, in terms of network resources and rapid introduction of new network level services. IoT controllers are specialized for the deployment and maintenance of IoT services and applications. They differ from the SDN controller in that they are service- or application-specified and each service provider or application may have a provider- or application-wide IoT controller. The IoT controllers can have a global network view with the help of the SDN controller. Depending on the scale of IoT applications, one central or multiple distributed IoT controller can interact with the SDN controller.

The infrastructure plane consists of routers, gateways, sinks, and IoT devices. Routers and gateways are responsible for forwarding data in the networks. In addition, gateways may store or cache local data, or process data under the instruction of controllers. Moreover, some control logic and functions can also be installed on the gateways.

The knowledge plane consists of IoT application and global network data. Data and knowledge related to the global network states is used by controllers to generate flow tables controlling data forwarding in routers and gateways in the infrastructure plane. IoT application data is generated from IoT devices and applications, including geo-spatial and time series information. Diverse data processing, analysis and learning methods can be used in the knowledge plane to extract useful data and knowledge and feed them to other planes.

The knowledge plane provides data and knowledge to the M&A plane, control plane and infrastructure plane through the “knowledge-bound” API. Since the goals and functions of the latter three planes are different, different types and levels of data expression, abstraction and content can be accessed by the three planes in different ways and for different purposes. The knowledge plane can adjust the content of data it obtains and the corresponding algorithms for processing them according to the needs of the three planes. For example, some real-time raw network data can be accessed by the SDN controller to configure routers, whereas concise abstract knowledge should be given to IoT controllers to make decisions on device management and task scheduling, etc., for IoT devices. In addition, according to the requirements from an IoT service provider, large amounts of data can be provided to it, but only the data related with this IoT service provider. Data for IoT device configurations can be provided to the IoT devices or gateways directly, depending on the capability of IoT devices. With the help of the knowledge plane, the other three planes will become intelligent and enable decision-making and self-adaptation to the environment.

Considering the heterogeneity of IoT devices and the scalability of the system, the IoT devices can be programmed in three ways, namely (i) through a logically centralized IoT controller, (ii) through a gateway physically close to the IoT device, which can be programmed by an IoT controller, and (iii) through a program installed on the IoT device by the IoT controller. We refer the program to IoT-proxy, which functionally belongs to the control plane and communicates with IoT controllers to program gateways and IoT devices. Thus, an IoT device may contain three functional modules: an IoT-proxy, programmable networking interfaces for sending and receiving data, and device-specific functions. Depending on whether IoT proxies are installed in the IoT devices, there are three basic types of IoT devices and the corresponding ways to program the devices to realize flexible IoT services:

(1) Non SDN-enabled devices, which cannot be installed as IoT proxies. They are connected to a router of the SDN-enabled network directly or through a sink. The sink is responsible for aggregating and caching the data obtained from or sent to IoT devices and performs simple processing, such as eliminating redundant data. The sink does not have control functions. In this case, all IoT related control functions are implemented at the IoT controller. These types of devices, including the sink, cannot be programmed.

(2) Pseudo SDN-enabled IoT devices, which are not installed IoT proxies, however, they can be reconfigured or programmed. In this case, the devices are connected to the SDN network through a gateway in the SDN-enabled network. The gateway supports more complex tasks, including configuring functions and simple control functions apart from forwarding data, and can also be programmed by the SDN or IoT controller.

(3) SDN-enabled devices, which are installed as IoT proxies with certain local control functions and can connect to the network directly and be programmed by the controllers directly.

Practically, which types of devices should be used are application and service provider dependent. Trade-off among the latency and overhead of the control, the scalability of controllers, the cost of IoT devices and the security should be considered. In general, an SDN-based IoT architecture supported by knowledge should have the following characteristics:

First, IoT application-specific controllers in the control plane, to program the IoT applications and services. Thus, the resource usage of the whole IoT network can be optimized together with the SDN controller. The separation of IoT controllers and SDN controller also makes the network easy to deal with the application-specific control, and at the same time maintain the network-wide information, overcome the resource underuse problem brought by the isolation of IoT applications and increase the flexibility of the network resource usage.

Second, network- and application-specific knowledge fed to the control, infrastructure, and management/administration plane through the “knowledge-bound” API. This can increase the intelligence and autonomy of the three planes in decision makings and makes the network well adapted to the environment, including the behaviors of IoT devices and applications. Furthermore, the application-specific programmability can be implemented down to the infrastructure plane, which increases the flexibility of the network, and reduces the service provisioning time.

Third, IoT proxies. This enables IoT control functions to be implemented in different ways through the interactions between IoT controllers and IoT proxies, which can realize the device-aware interface and programmability, optimize the placement of controllers, and maintain the scalability and performance of the network.

In addition, many data analytic methodologies and algorithms can be used for building, deploying, and applying the knowledge plane. They should be robust, lightweight, and be able to capture dynamic arrivals of data. Online machine learning algorithms could be a good choice when IoT application data is dynamic. Models are updated continuously as new data arrives, reducing storage requirements and is adaptive to applications. Distributed machine learning algorithms are also good candidates when data sources are big and geo-distributed by greatly reducing the computation burden at each node. Machine learning models relevant to the knowledge plane include Long Short Term Memory (LSTM), convolution networks, and Restricted Boltzmann machine. For example, by using the feature of long and short term memory, an improved LSTM algorithm [67] can be used in the knowledge plane to predict the IoT data traffic at the gateways according to the historic IoT data traffic, and send the information to an SDN controller. The SDN controller can then configure the bandwidth at the corresponding gateway dynamically. Moreover, alternative training strategies can be used to improve model accuracy, such as federated learning, decentralized deep learning, communication-efficient learning, and distributed optimization.

### 4.4. An Example Application—Industry IoT

**Figure 3:**
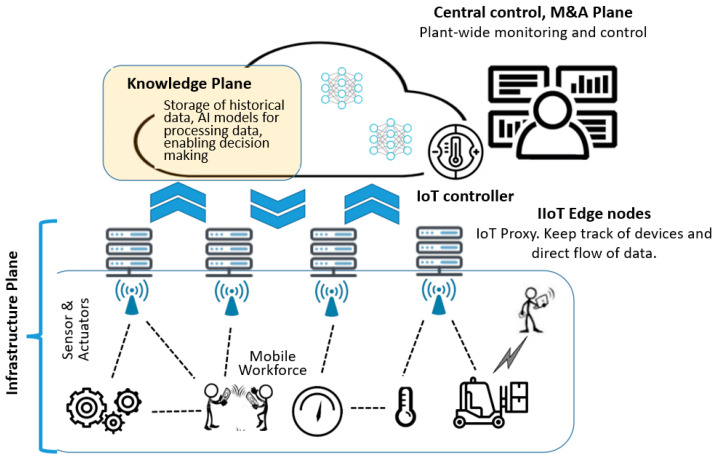
Example application where a mobile workforce is supported in an Industry IoT (IIoT) scenario. Huge amounts of data generated by equipment need to be collected both for long-term storage and analysis at a low resolution and for mobile workers used locally at high resolution (near real-time). An IoT-proxy can be installed in the IIoT Edge nodes to control the data transmission frequency to the clouds/control center. An IoT controller can be realized in the control center, and the cloud services can be realized in the knowledge plane.

Figure 3 illustrates an example of Industry IoT (IIoT) [68,69] where the enhancement of SDN-based by knowledge would be beneficial. In this example, the overall production monitoring/management and historical data archiving uses central control rooms-collected data. When operational issues are detected, diagnostics and local maintenance can be executed and performed by mobile workers, who rely on information from production system, both for their own safety and to solve problems within the plant. Historical data (at lower resolution) is relied upon by the workers to pinpoint the root causes of the problems, as well as the use of real-time data flows to assess the current state of the equipment.

Massive amounts of sensors and actuators are connected in industrial processes, and there exists at least one dataflow between each sensor or actuator and the control system. These data flows are often periodic and typically have a timeframe in the order of milliseconds between each data transmission. The collection of historical data for analysis, typically requires measurements to be fed from process controllers to a central historian server at time intervals in the order of one second. This implies that high transmission frequency data flows with small amounts of data per transmission are to be found close to the actual process used for control purposes, while analysis and statistics is performed on less detailed information which is stored on the historian servers.

The above application scenario can be realized easily and efficiently by using the IoT architecture enhanced by knowledge-driven SDN. As shown in Figure 3, an IoT-proxy can be installed in the IIoT edge nodes. It can keep track of the different industrial devices and provide a means for directing the flow of data so that data going to a central control room and archival services can be sent at a lower priority, and not conflicting with more urgent time-critical data. At the same time, distribution of high resolution data needed by (possibly multiple) operators on the factory floor can be distributed locally without wasting global network resources or imposing unnecessarily long latencies. Moreover, the function of the central control can be realized as an IoT controller. On the one hand, it has a plant-wide (i.e., the application level) control. On the other hand, it can coordinate, for example, bandwidth and computing resources with the network operator through the SDN controller to guarantee the performance of the data transmission and processing. The cloud services can be realized in the knowledge plane. The analysis results can be accessed by the IoT controller (i.e., the central control), which further instructs the IoT-proxy in the IIoT edge nodes to track the devices. In addition, according to the privacy and security protection strategy, the historical data can also be used by other similar IIoT scenarios as a resource.

## 5. Challenges and Future Work

The knowledge-driven SDN-based IoT endows a wide and deep programmability to the networks and IoT applications, which enhances the IoT networks and services in a unified and horizontal way, providing a flexible and intelligent deployment model for IoT services. To take advantage of the approach, the following issues should be further investigated to deal with the challenges brought by both obtaining and using knowledge and the use of SDN technology.

- API among different planes. A knowledge-bound API will be introduced in the approach due to the introduction of the knowledge plane. Since the M&A, control and infrastructure plane have different levels of computation and decision-making capabilities, they may require different levels of knowledge abstractions. Hence, different types of knowledge-bound APIs should be defined. In addition, due to the introduction of IoT controllers, the northbound and southbound APIs should also be extended. The constraints of IoT devices and the introduction of programmability on the devices (e.g., through IoT proxies) require also a new and efficient southbound API.
- Intra-plane communication. To realize the efficient information exchange among IoT controllers, the SDN controller and IoT proxies, the intra-plane communication should be investigated.
- Performance and security. When introducing SDN, the critical IoT performance metrics such as latency, energy efficiency, throughput, scalability, and packet lost and jitter need to be considered. Moreover, service availability and reliability are important concerns. In addition, the SDN-IoT integration environments open up new security risks, which should be addressed.
- Efficiency. Knowledge and IoT controllers introduce management overhead. Hence, the trade-off among flexibility, intelligence and application-specific control should be considered.

In addition, the methods and algorithms in the knowledge plane should also be investigated, taking the characteristics of IoT into consideration. For example, the data analysis and learning algorithms should be application-specific. The traffic monitoring and performance measurement should consider carefully the influences of large amounts of heterogeneous IoT data. Although the IoT proxies can simplify greatly the realization of collective intelligence of IoT, the size and scope of the IoT proxies in terms of the collective intelligences should be designed carefully.

Moreover, new challenges may be put forward to the network management after introducing the IoT controllers and knowledge plane. How to realize efficiently the global network maintenance and policy control should be further studied.

## 6. Conclusions

Leveraging the data generated by IoT applications, SDN can enhance the control and management of IoT in terms of flexibility and intelligence. Based on this consideration, in this paper, we performed a comprehensive review of the evolution of SDN and IoT and discussed the benefits and challenges brought by integrating SDN and IoT with the help of IoT data. Based on reviewing the existing work regarding big data for SDN and IoT, we analyzed the perspectives of knowledge-driven SDN for the enhancement of IoT in flexibility, intelligence, and application-specific control of IoT networks through a concrete knowledge-driven SDN-based IoT architecture (KN-SDN-IoT) and an Industry IoT application. By introducing a knowledge plane together with the knowledge-bound API and IoT proxies in the devices, different levels of application- or IoT provider-specific control can be realized, and IoT devices and data can be used efficiently, similar to other types of network resources. Other challenges faced by the current IoT systems, such as collective intelligence, isolated islands of deployment, etc., can be easily solved. In addition, the characteristics of the SDN-based IoT supported by knowledge were elaborated, and its relationship with the AI-based cloud and edge computing were discussed. Finally, challenges and future research related with enhancing the IoT with knowledge-driven SDN were discussed.

## Figures and Tables

**Table 1:** Comparison of surveys about SDN and IoT.

Reference	Year	Domain	Contributions
[14]	2020	Network virtualization for SDN-based IoT	A comprehensive survey on architecture, security and management solutions of NFV (network function virtualization) for IoT and SDN-based IoT.
[15]	2019	SDN for edge computing, which used for serving IoT	A survey on how SDN can facilitate the management and operations of edge servers, and how SDN can provide programmable interfaces to IoT devices.
[16]	2018	SDN for network management and IoT	A brief survey of how network management and IoT network management can be improved by SDN
[3]	2018	SDN and fog computing for IoT	A review of SDN and fog computing-based solutions to overcome the main challenges of IoT, such as large amount of data, security, high network scale etc.
[17]	2018	Security	A comprehensive analysis of security features introduced by NFV and SDN for IoT systems.
[18]	2018	SDN for IoT architectures.	A brief survey of solutions about SDN-assisted IoT architectures.
[19]	2017	SDN for security and performance of IoT	A brief survey on security and performance improvement by using SDN for IoT
[20]	2017	SDN-based IoT solutions	An analysis of the existing solutions of SDN-based IoT, including SDN-based cellular network, IoT management framework, IoT security solutions etc.
[1]	2017	SDN technologies for IoT	A comprehensive survey of how different SDN technologies can fulfill the requirements of IoT, from the viewpoint of edge, access, core, and data center network.
[2]	2016	SDN and NFV for IoT	Survey on the combination of SDN and NFV for wireless sensor networks and for IoT, including architectures and application.
[21]	2015	Integrating SDN and IoT	An early survey of SDN and its application in wireless networks (SDWN). The challenges in security and scalability of SDWN for IoT are surveyed.
[22]	2014	SDN for IoT applications	An early survey of SDN and its opportunities in the development of IoT applications.

**Table 2:** Benefits of SDN to IoT.

Benefits	Metrics	Example Practices
**Improving IoT management through SDN**	Resource (bandwidth, computation, storage)	The controller manages and assigns the heterogeneous network and device resources to heterogeneous IoT tasks by using information collected from the network environment [25] (SDN@home), and SDN-based traffic engineering [26].
	Device	Controllers have policies to manage sensor nodes, to choose the right wireless connectivity, and to control the IoT device.
	Application	Through a shared physical infrastructure, the same set of sensor nodes can support multiple applications from multiple developers. The network behavior is customized through software.
	Infrastructure	IoT infrastructure is built through SDN and NFV [27] (SDIoT). Specific service requirements are translated by a central controller into network requirements for, e.g., data rate and packet loss. Mechanisms, e.g., network calculus, are used to model the multi-network environment and algorithms are used to schedule flows.
	Energy usage	Duty cycles, a sleep-scheduling mechanism, and in-network data aggregation for low rate WPAN (LR-WPAN) are realized through an SDN controller [28]. Each computation is completed in the controller rather than in the sensors themselves and there is no broadcasting between sensor nodes.
	Users/ owners	OpenFlow-based system supports user monitoring and management and Internet access control. Multiple service providers can share a common infrastructure. The system supports verification policies and business models for cost sharing in the smart home environment.
	Policy	Software-defined WSN to tackle problems, such as rigidity to policy changes and difficulty in management [29].
	Mobility management	A distributed hashing-based overlay structure realizes flow scheduling and mobility management [30] (UbiFlow).
	Quality of Service (QoS)	Specific service requirements are translated into network requirements by a central controller to optimize the end-to-end performance, e.g., data rate and delay for each flow. ISP can expose some control to users to provide QoS for specific devices and applications [31]. SDN controller incorporates and supports commands to differentiate flow scheduling over task-level, multi-hop, etc., and exploits genetic algorithms to optimize the usage of currently available IoT network opportunities [32].
**Improving IoT performance & service provisioning through SDN**	Link reliability/resilience	Alternative paths are calculated based on SDN’s global view; OpenFlow-based systems use restoration and protection mechanisms [33].
	IoT service provisioning	Integrated Cloud/Fog and network resources are orchestrated to provide network connectivity between IoT gateways; virtual machines are deployed at edge nodes [34].
	Edge computation offloading	Programmable orchestrator enables fine-grained offloading of IoT computing tasks [35] (FADES).
	Time-constrained big data transfer scheduling	Support dynamic data scheduling through SDN controllers in order to support time-constrained data transmission for smart cities scenario [36].
	Scalability and mobility of IoT	Virtualize the IoT gateway to make it possible to be dynamic, scalable and elastic in an IoT environment with NFV implementation [37].
	CPU use/network expansion	CPU can be optimized and network expansion can be realized through, e.g., different types of routing assisted by SDN controllers [38].
**Enhancing IoT architecture through SDN**	SDN-based wireless network [7]; SDN-based sensor networks [39]	Abstract service, network and sensing layers correspond with the SDN architecture [37]: physical infrastructure layer, control layer, and application layer are set for urban sensing. SDN controller realizes intelligent scheduling algorithms to provide QoS-aware routing. Scalability problems are solved through several SDN domains, each with a controller; heterogeneous IoT flows are handled. Making sensor nodes programmable as finite state machines [40]
	SDN for industry IoT	Enhance the interoperability of IoT devices for Industry 4.0 by considering the heterogeneity of communication protocols and data formats, etc. through an open-source software architecture solution [41].
	SDN for vehicular network	Hybrid, hierarchical SDN-based architecture for vehicular networks, to mitigate the connectivity loss between vehicles and central SDN controller [42], optimize operating expense [43] and radio resource [44], and increase security [45].
	SDN-based IoT architecture for horizontal services	A layered IoT architecture with multiple SDN controllers in order to provide general horizontal IoT services [46].
	SDN-based IoT for smart grid	Cloud-SDN and decentralized Fog-SDN architectures, to schedule users’ requests in a real-time way and to supervise communications between microgrids controllers [47].
**Enhancing IoT** **security and** **privacy through SDN**	Policy-based security control	A security module (middlebox-guard) is used, which can put security policies in the most appropriate locations with the help of SDN controllers [48].
	Detect and mitigate DoS and DDoS [49]	To detects the DoS and DDoS attacks according to the entropy values in the SDN controller. Implemented using OpenState, an extension to current OpenFlow in a stateful SDN data plane [50].
	Wireless isolation for IoT;	The controllers analyze and approve connections and traffic flows in the network. Once a connection is authorized, the corresponding flow tables are installed in the switch. Fine-grained control of flows enhances security and privacy [51].
	Identity-based authentication;	The specific identity formats used by different protocols are mapped to a shared identity via the SDN controller, where a trusted certificate authority is implemented [17].
	Mitigate traffic analysis and data gatheringattacks;	Both header and payload are encrypted. A simple broadcast routing protocol aided by the SDN controller is used to solve the header encryption problem [52].
	Role-based security control	Intrusion controller, key controller, and crypto controller [53].

**Table 3:** Requirements on SDN driven by IoT.

	Legacy SDN	Problems in IoT and IoT’s Requirement	Requirements on SDN
**Controller’s functions**	Traffic forwarding; device configuration; resource management	Data collecting, processing, and distribution also need to be controlled; multiple types of connectivity, multiple protocols and various device capability need to be supported; scalability problem; data, software and security also need to be managed.	Multi-metric and self-adaptive routing and forwarding schemes; flexible definition of rules; connectivity-, reliability-, latency-, and energy-awareness; intelligent and collective use of data collected from various devices; dealing with heterogeneity and scalability.
**Controller’s placement**	Logically centralized	Longer delay caused by wireless transmission; decreased performance due to large numbers of heterogeneous nodes with different networking capabilities.	Optimize placement in the designed network topology, considering data processing and high scalability of device and networking protocols.
**Network elements and protocols**	Routers in wired network; homogeneous with some difference in capacity; IP protocol, limited MAC/physical layer protocols	Mobile routers/nodes; resource/energy constrained IoT devices should be considered; large differences in capability of network nodes; a variety of MAC and physical layer protocols.	Mobility-aware control (incl. changes in topologies); adaptation and/or mapping functions; function abstraction and mapping; very low overhead protocol; data/demand/application-driven protocols.
**Interfaces**	South-bound API and north-bound API OpenFlow	Heterogeneous devices, wireless transmission, bandwidth and energy constraints.	Device-aware interfaces: abstract IoT’s behaviors for decision-making of SDN.
**Applications**	Flow-based; limited types of applications	Flow and packet-based; very diverse delay requirements, ranging from emergency or time critical to very delay tolerant applications; millions of applications from different domains need to be supported.	Application-specific consideration: average service rate of controller, average arrival rate of initiation requests, and the path inflation factor depend on the distance of the distributed controllers, channel capacity, flow size, and the network topology.
**Architecture**	Well-known hierarchical architecture with standardized interfaces	Wide area coverage, data aggregations at the edge; with multi-hop relaying; relaxed requirements for handover and roaming support.	Optimized controller placement; edge computing; dynamic and self-adaptive interworking mechanisms and policies.

**Table 4:** Current work about data for SDN and IoT.

Work	Data-Driven SDN [57,58]	Big Data of IoT [54,55,56]
Aimed network	ICN (Information centric network) CDN (content delivery network)	IoT
Method	Introducing data plane or data engine	Using SDN to monitor network states and resources
Goals/Solved problems	Intelligent decision-making	Efficient distribution of IoT functions/services
Benefits for IoT	To a certain degree, can be used to realize application-specific control, data management, security and privacy	Application-specific control, device/data management

**Table 5:** Difference between cloud, edge computing and knowledge-drive SDN for IoT.

Functions	Cloud Computing with AI	Edge Computing with AI	Knowledge-Driven SDN for IoT
Data colleting	Yes	Yes	Yes
Usage of intelligence	Decision-making for applications	Decision-making for applications and devices	Control of routing, resource optimization, decision-making for applications and devices
Location of intelligence (algorithms)	Cloud server	Edge nodes	SDN and IoT controllers, IoT-proxy, database; forwarding plane through API
Programmability/ configurability	Cloud server (manually through software update)	Edge nodes (manually through software update)	Routers/Switches, IoT devices
Scalability	No	To edge nodes	To IoT devices
